# Metagenome of Gut Microbiota of Children With Nonalcoholic Fatty Liver Disease

**DOI:** 10.3389/fped.2019.00518

**Published:** 2019-12-20

**Authors:** Yuzhen Zhao, Jianli Zhou, Jiaqi Liu, Zhaoxia Wang, Moxian Chen, Shaoming Zhou

**Affiliations:** ^1^Division of Gastroenterology, Shenzhen Children's Hospital, Shenzhen, China; ^2^Shantou University Medical College, Shantou, China

**Keywords:** nonalcoholic fatty liver disease, gut microbiota, metagenome, children and adolescents, obese

## Abstract

**Aim:** To investigate the intestinal flora of nonalcoholic fatty liver disease (NAFLD) in Chinese children and adolescents using metagenomic approach.

**Methods:** All participants underwent magnetic resonance spectroscopy (MRS) to quantify liver fat content. Hepatic steatosis was defined as MRS proton density fat fraction (MRS-PDFF) >5%. A total of 58 children and adolescents were enrolled in this study, including 25 obese NAFLD patients, 18 obese non-NAFLD children, and 15 healthy children. Stool samples were collected and analyzed with metagenomics. We used Shannon index to reflect the alpha diversities of gut microbiota. Wilcoxon rank sum test and Kruskal-Wallis test were performed to evaluate alpha diversities between groups. At last, the differences of gut microbiota composition and functional annotations between obese with and without NAFLD and healthy children were assessed by Kruskal-Wallis test.

**Results:** Significant differences in gut microbiota composition and functional annotations among three groups of children and adolescents have been observed. Deep sequencing of gut microbiota revealed high abundance of phylum *Proteobacteria* (*Gammaproteobacteria*) in obese NAFLD patients, comparing with the control group. Overall, obese children without NAFLD had less abundant *Helicobacter* and *Helicobacter pylori*. Compared to the control group, in obese children with NAFLD, the abundance of *Bacteroidetes* (*Alistipes*) were significantly reduced. *Faecalibacterium prausnitzii* was the only species representing a difference between obese children with and without NAFLD. There were not significant differences in terms of alpha diversity among three groups. Functional annotations demonstrated that several pathways were differentially enriched between groups, including metabolism of other amino acids, replication and repair, folding, sorting, degradation, and glycan biosynthesis and metabolism.

**Conclusion:** Significantly differences are observed in gut microbiota composition and functional annotations between obese children with and without NAFLD in comparison to the healthy children group. The characteristic of gut microbiota in this study may contribute to a further understanding the gut-liver axis of pediatric NAFLD in China.

## Introduction

Nonalcoholic fatty liver disease (NAFLD) has emerged as one of the most common chronic liver disorders in children and adolescents, affecting 5–10% of the general child population, and 28–41% of obese children (1). The term NAFLD is also known as a broad spectrum ranging from simple steatosis, nonalcoholic steatohepatitis (NASH), to hepatic cirrhosis. In addition to liver-related disorders, NAFLD is also revealed as a risk factor of chronic kidney disease, cardiovascular diseases, obesity, and diabetes (2–4). What is more, 20% of NASH cases may progress to hepatic cirrhosis, and eventually liver failure (5). Therefore, NAFLD is of great importance on public health, especially in children and adolescents.

The human intestinal tract is the main habitat of microbiota, with ~10^14^ bacteria existing, which comprises of 100 times more genes than humans. As a group of symbiotes, gut microbiota play an important role in metabolic and immune balance of human beings. First, gut microbiota participate in digestion and absorption of nutrients and production of vitamins and minerals. Second, gut microbiota can affect the production of intestinal hormones such as glucagon-like peptide-1, thereby affecting the metabolism of the host (6). In recent research, we learn that gut microbiota is closely related to the occurrence and development of NAFLD through Gut-liver axis (7, 8). Increasing evidence suggests that gut microbiota connect with hepatic steatosis in several ways (9–11): (1) it affects the appetite signal of the host; (2) it can also increase energy extraction from the intestine; (3) the metabolism of bile acids change, therefore affecting fats and lipid-vitamins obtained in the intestine; (4) the metabolism of choline is affected; (5) it also contributes to increased inflammation in host organisms; (6) intestinal bacterial overgrowth and intestinal permeability increase will lead to bacteria translate into the systemic circulation and endotoxemia.

As early as 2004, Backhed et al. first connected gut microbiota with obesity and NAFLD (12). They found that gut microbiota could not only affect the absorption and storage of energy, but also stimulate the production and infiltration of triglycerides in hepatocytes. Mouzaki et al. (11) diagnosed NAFLD with liver biopsy and further detected the composition of gut microbiota. They demonstrated that gut microbiota in healthy controls were varied from patients with simple fatty liver disease and NASH. The *Bacteroides* in NASH patients were significantly lower compared to that in simple fatty liver disease patients and healthy controls, and were independent from diet and BMI. While Raman et al. (13) and Li Fan et al. showed that compared with healthy controls, the differences in intestinal flora of NAFLD mainly occurred at family and genus levels, of *Firmicutes* (14).

In recent years, with increasing prevalence of obesity and NAFLD in children and adolescents, drawing more attention of researchers to these populations. Zhu et al. performed 16S RNA sequencings to analyze the composition of gut microbiota in Caucasian children in a controlled diet with NASH, obesity, and healthy controls in the United States (15). They suggested that alcohol-producing bacteria *Escherichia* was significantly increased in NASH children. Comparing with healthy controls, children with NASH and obesity had increased *Bacteroidetes* and decreased *Firmicutes*. The differences of gut microbiota between NASH patients and obese children were exhibited in *Proteobacteria, Enterobacteriaceae*, and *Escherichia*. Michail et al. (16) conducted metagenomics and proteomics to examine the composition, function, and metabolism of intestinal flora in obese children with and without NAFLD against the healthy children controls. *Gammaproteobacteria, Prevotella* and ethanol were observed to have a significant increase in NAFLD patients. In addition, there was an increase in the pathways involved in energy metabolism and lipid metabolism in children with NAFLD, compared with the healthy controls. Researchers in Italy (5) analyzed the gut microbiota in children with simple fatty liver disease, NASH, obesity, and healthy children but got a different result. Compared with healthy groups, *Actinobacteria* were increased in NAFLD children, while *Bacteroidetes* were decreased, which was contrary to the research by Zhu et al. (15). There was no significant difference in gut microbiota composition among the groups of children with simple fatty liver disease, NASH, and obesity, which was consistent with the previous study by Zhu et al.

Researchers have demonstrated that gut microbiota of NAFLD patients could undergo a compositional and functional change, but the differences of gut microbiota in obesity, NAFLD, and healthy children have no consistency. Furthermore, studies are rarely reported in the case of Chinese pediatric NAFLD. Therefore, the present study used metagenome to clarify the composition and function of gut microbiota in obese children with and without NAFLD against healthy children in China in order to unravel the relationship of liver-gut axis and its function in children NAFLD.

## Materials and Methods

### Subjects

The research was approved by the Human Ethics Committee of Shenzhen Children's Hospital (Supplemental Document Ethic Committee Approval). A total of 58 participants were recruited in the study during May 2017 to July 2018, including 43 obese [body mass index (BMI) above age- and gender-specific 95th percentile] and 15 healthy controls. The average age of participants is 13.7 years, ranging from 9 to 17 years. In view of their diagnosis in our previous study (17), children and adolescents were stratified into obesity with (*n* = 25) or without (*n* = 18) NAFLD, and matched healthy controls (*n* = 15). All subjects with antibiotics or probiotics history in the past 3 months were exclude. All authors of this paper had access to the study data and reviewed and approved the final manuscript.

### Magnetic Resonance Spectroscopy (MRS)

Children and adolescents underwent single-voxel MRS scanning using 3.0 T MR unit (MAGNETOM Skyra, Siemens Healthcare, Erlangen, Germany) in accordance with previous research (17). Hepatic steatosis is defined as MRS proton density fat fraction (MRS-PDFF) > 5%.

### Fecal Sample Collection, DNA Extraction and Metagenomic Sequencing

Before collecting, methods and notes were explained by researchers. Samples were collected using a sterile kit and frozen at −80°C immediately until detection.

All stool samples were analyzed in Beijing Genomics Institute (BGI, Shenzhen, China). Briefly, DNA was isolated from 58 fecal samples and an average of 5.92 Gb of sequence was obtained from each subject. All DNA fragments were purified by QIA quick PCR Purification Kit (Qiagen) during library construction. Agilent 2100 Bioanaylzer and ABI StepOnePlus Real-Time PCR System were used to qualify and quantify the sample libraries. The qualified libraries were then sequenced using Illumina HiSeq System.

### Metagenomic Analysis

In order to acquire accurate sequences, we removed sequences with a 90% similarity to the human genome. Then we assembled all samples to obtain reads which were more than 300 bp for further analysis. With CD-Hit software (18), genes were combined and clustered. Finally, we got 2,053,172 non-redundant genes. All these genes were blasted against with Kyoto Encyclopedia of Genes and Genomes (KEGG) databases using the Diamond software (19) to obtain function annotations. The taxonomic composition was performed using MEGAN software (20) based on NR databases. Alpha diversity was calculated using Shannon index based on species profile.

### Statistical Analysis

All data were statistically analyzed using SPSS 22.0 software and R environment. Numerical variables were firstly tested for normality. Age and BMI between three groups were compared by independent-samples *T*-test. The Chi-square test was performed for gender comparison between groups. The differences between groups in taxonomic composition and function annotations were determined using Kruskal-Wallis test. Alpha diversity was calculated based on Shannon index. Wilcoxon Rank-Sum test or Kruskal-Wallis test were used for diversity differential analysis, depending on group numbers.

## Results

### Clinical Characteristics

A total of 58 participants were enrolled in the present study with an average age of 13.7 ± 1.8 years (range 9–17 years). According to MRS examination, hepatic steatosis was diagnosed with MRS-PDFF > 5%. We divided all subjects into three groups based on liver fat content and BMI. Among them were obese NAFLD patients (*n* = 25), obese patients (*n* = 18), and healthy controls (*n* = 15). There was no difference in gender and age among these three groups. The basic information of these three groups is summarized in Table 1.

**Table 1:** Characteristics of all subjects.

	**Obese NAFLD (*n* = 25)**	**Obesity (*n* = 18)**	**Health controls (*n* = 15)**
Male	19 (76.0%)	12 (66.7%)	10 (66.7%)
Female	6 (24%)	6 (33.3%)	5 (33.3%)
Age (years)	14.1 ± 2.1	13.9 ± 1.3	13.7 ± 2.0
BMI (kg/m^2^)	30.6 ± 3.4^*^	28.6 ± 1.8^*^	20.2 ± 1.9
MRS-PDFF	15.5% ± 8.8%	2.8% ± 1.2%	1.3% ± 0.26%

* *P < 0.05 versus healthy controls*.

### Taxonomy Comparison of Gut Microbiota at Phylum Level

**Figure 1:**
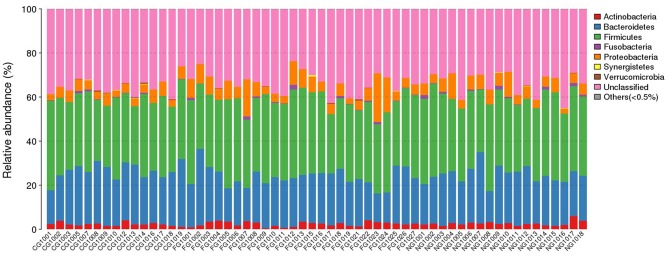
Phylum distribution of gut microbiota of all subjects. The distribution of bacterial phyla (abundance >0.5%) of each individual is presented as bar chart in terms of percentage weight.

**Figure 2:**
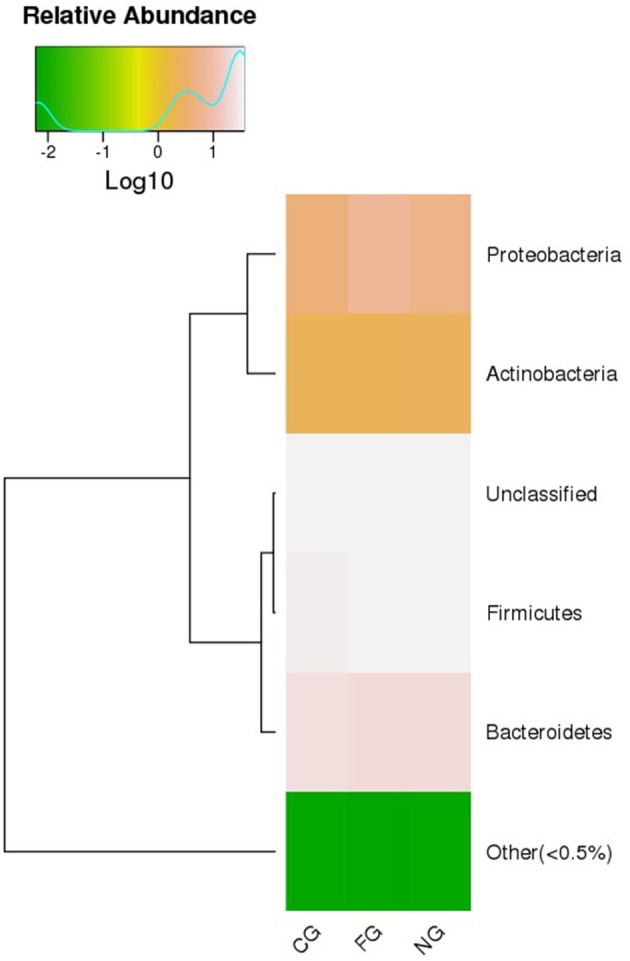
Heat map of gut microbiota between groups at phylum level. Group comparison and clustering of major bacteria phyla and their relative abundance. CG, healthy controls; FG, obese NAFLD group; NG, obese non-NAFLD group.

Taxonomic composition of all subjects was analyzed at phylum, class, order, family, genus, and species level. To obtain an accurate taxonomic change in all samples, abundant levels with <0.5% average abundance were classified into others. We observed significant differences between obese patients with and without NAFLD and healthy controls. At phylum level, we found that *Firmicutes, Bacteroidetes, Actinobacteria*, and *Proteobacteria* were the dominant phyla in all samples (Figure 1). Comparing with the healthy controls, the abundance of *Proteobacteria* was statistically increased and *Bacteroidetes* was decreased in obese NAFLD children. In contract, *Firmicutes* and *Actinobacteria* did not show large variations in three groups of children (Figure 2).

### Taxonomy Comparison of Gut Microbiota at Class, Genus, and Species Level

Within phylum *Firmicutes*, class *Negativicutes*, genus *Phascolarctobacterium*, and species *Phascolarctobacterium succinatutens* exhibited a significant increase in obese NAFLD children, compared with the healthy controls (Supplemental Table 1). In contrast, genus *Lactobacillus, Oscillibacter*, and *Ruminiclostridium* showed a strong decrease in obese NAFLD patients. Additionally, we observed that *Faecalibacterium prausnitzii* was the only species representing differences between obese children with and without NAFLD. The Pathogenic genus *Clostridium* showed no statistical difference among these groups.

Decreased abundance of *Bacteroidetes* was mainly due to the decreased abundance in class *Bacteroidia*, genus *Alistipes*, and *Paraprevotella* in the obese children with or without NAFLD (Supplemental Table 1). Besides, at species level, we found that the abundance of *Bacteroides clarus* and *Odoribacter splanchnicus* were reduced in obese children groups with and without NAFLD. Interestingly, *Parabacteroides johnsonii* only exhibited a decrease in the obese NAFLD group, compared with the healthy group.

The increased representation of *Proteobacteria* in obese NAFLD group was mostly explained by the increased class *Gammaproteobacteria*, genus *Klebsiella, Kluyvera*, and species *Klebsiella pneumoniae* and *Kluyvera ascorbata* (Supplemental Table 1). It is worth noting that genus *Helicobacter* and species *Helicobacter pylori* were present and decreased in obese non-NAFLD children, compared with the healthy control group.

Furthermore, it is noteworthy that there were not any differences in phylum *Actinobacteria* in the three groups of children, which was in contrast to previous studies (15).

### Ecological Diversities of Gut Microbiota

In the present study, we used Shannon index to assess the Alpha diversity of the community. Three groups of subjects did not demonstrate statistical difference in diversity (Table 2).

**Table 2:** Comparison of Shannon-index between groups.

**Group**	**Statistical method**	***P*-value**
FG vs. CG vs. NG	Kruskal-Wallis	0.3
FG vs. NG	Wilcoxon Rank-Sum	0.6
FG vs. CG	Wilcoxon Rank-Sum	0.1
CG vs. NG	Wilcoxon Rank-Sum	0.3

*CG, healthy controls; FG, obese NAFLD group; NG, obese non-NAFLD group*.

### Functional Annotations of Gut Microbiota

**Figure 3:**
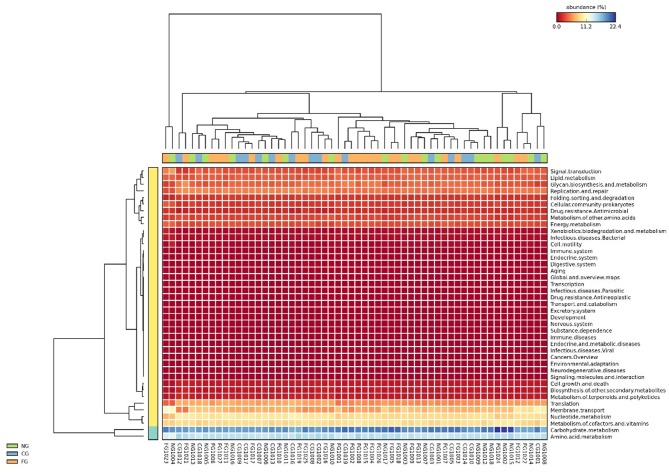
Heat map of gut microbiota KEGG functional annotations. The KEGG pathway annotation was carried out for each individual sample. The abundance of each KEGG term was then clustered to represent as a heatmap.

**Figure 4:**
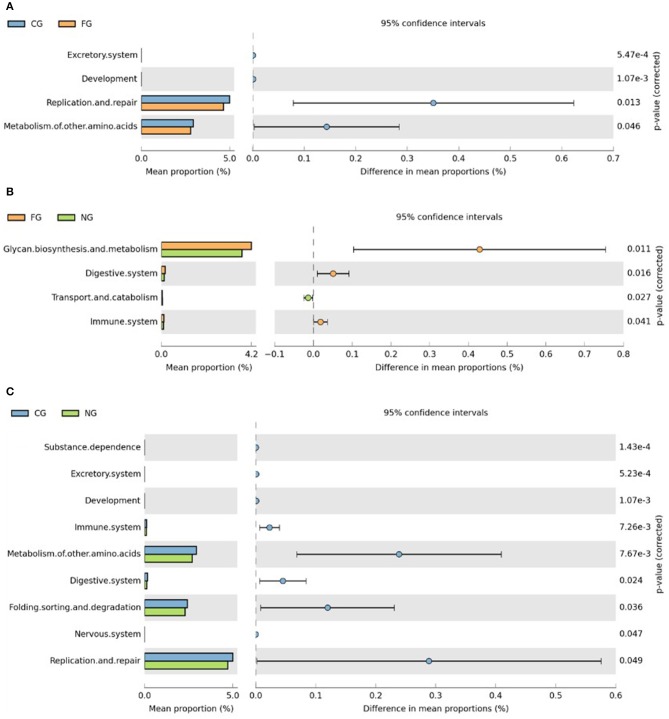
KEGG category comparisons among three groups of children. **(A)** Differences between obese NAFLD and healthy controls in KEGG functional annotations. **(B)** Differences between obese patients with and without NAFLD in KEGG functional annotations; **(C)** Differences between obese non-NAFLD and healthy controls in KEGG functional annotations. CG, healthy controls; FG, obese NAFLD group; NG, obese non-NAFLD group.

Subsequently, we further assessed functional annotations of gut microbiota via Diamond software against pathway information from the KEGG database. In total, by using Wilcoxon rank-sum test, we observed 923 KEGG orthologies (KOs) in three groups (Figure 3). Compared to healthy controls, pathways such as replication and repair and metabolism of other amino acids in KEGG levels 2 were significantly decreased in obese children with and without NAFLD (Figures 4A,C). The folding, sorting, and degradation pathway decreased in obese patients as well, in comparison to the healthy group. Furthermore, pathways like the digestive system, immune system, glycan biosynthesis, and metabolism showed considerable differences between the two obese groups, with higher abundance in the group of obese NAFLD patients (Figure 4B).

## Discussion

In the present study, we have assessed gut microbiota of obese children with and without NAFLD in comparison to healthy controls. With metagenomic analysis, we learnt that microbial composition of three groups was significantly different at levels of phylum, class, genus, and species. At phylum level, obese NAFLD children exhibited increased *Proteobacteria* and decreased *Bacteroidetes*, compared to the healthy group, while *Firmicutes* showed no difference between groups. Dating back to 2006, Turnbaugh et al. (21) found that the ratio of *Firmicutes* to *Bacteroidetes* increased in obese mice. In their research, they came to the hypothesis that *Firmicutes* were a group of microbiomes relating to obesity. Genes related to the metabolism of fat and indigestible polysaccharides were detected in *Firmicutes*. In contrast, the population of *Bacteroidetes* is strongly increased in lean mice and humans, suggesting its representative role of body composition (22). The ratio of *Firmicutes* to *Bacteroidetes* also increased in our study. Thus, we postulated that the ratio of *Firmicutes* to *Bacteroidetes* could be a potential biomarker of NAFLD.

In *Bacteroidetes*, compared to the healthy group, genus *Alistipes* (associated with metabolism of plant cell wall polysaccharides and resistant starch) were decreased in obese NAFLD patients. It was in line with research by Zhu et al. (15) and Jiang et al. (23). In compensated and decompensated cirrhosis patients, the abundance of *Alistipes* decreased as well (24). Thus, *Alistipes* may be a group of beneficial microbiomes.

Within *Proteobacteria, Gammaproteobacteria* were observed to be increased in obese NAFLD patients in this study. *Gammaproteobacteria* are thought to be involved in choline metabolism and might be inhibited in high levels of choline (25). Besides, the relative abundance of *Gammaproteobacteria* is negatively correlated with liver fat content. However, a contrasting result was present in our study, which may due to the differences in age, religion, and diet of the test subjects. We hypothesized that dietary habits are related to the composition of gut microbiota. Therefore, we can restrict the diet in all subjects for further analysis. Furthermore, genus *Helicobacter* and species *Helicobacter pylori* of *Proteobacteria* were found to be differentially accumulated between obese and healthy children. Researchers in Korea (26) and China (27) found that *Helicobacter pylori* infection was closely related to the development of NAFLD based on large population research. Eradication of *Helicobacter pylori* could be a new method to deal with NAFLD. However, many studies do not find any connection between *Helicobacter pylori* infection and NAFLD (28, 29). Similarly, we did not observe significant changes between NAFLD patients and healthy children, either. Differently, in this study, we directly detected the *Helicobacter pylori* from stool samples, while most of the above studies detected *Helicobacter pylori* via serum antibody or urea test. Therefore, further study is needed to assess the relationship of NAFLD and intestinal *Helicobacter pylori*.

Although there was no significant difference in *Firmicutes* between three groups in this study. The abundance of probiotic *Lactobacillus* and anti-inflammatory species *Faecalibacterium prausnitzii* have been reported to be decreased in obese NAFLD children. Besides, the abundance of *Faecalibacterium prausnitzii* has been observed to be decreased in NASH patients (15, 30). To our knowledge, *Faecalibacterium prausnitzii* belong to beneficial microbiomes *Clostridium*, which can be developed into a potential kind of probiotic. The ecological imbalance of intestinal *Faecalibacterium prausnitzii* is closely related to inflammatory bowel disease, irritable bowel syndrome and type 2 diabetes (31, 32).

In the current study, ecological diversities of gut microbiota in three groups of subjects did not indicate differences. The same consequence was observed in study by Zhu et al. (15). Gut microbial alpha diversity between NASH patients, obese children and healthy controls were not statistically different. While Del Chierico et al. (5) conducted metagenomic analysis to assess gut microbiota of patients with simple fatty liver disease and NASH, obese, and healthy children. They found that the degree of alpha diversity was highest in healthy children, followed by obese children, NASH children, and simple fatty liver disease patients. Therefore, further study is necessary to analyze the ecological diversities in NAFLD.

Through functional annotations, we observed that several pathways were differentially enriched among these groups, including metabolism of other amino acids, replication and repair, folding, sorting, degradation, and glycan biosynthesis, metabolism and so on, whereas pathways of carbohydrate, lipid, and amino acid metabolism showed no differences. Further investigation of enriched gene clusters may be required to unravel the potential functional module related to obesity or NAFLD. In addition, researchers in Italy have found that urinary metabolomics of obese and NAFLD children are significantly different (33). Therefore, we can also analyze the urinary metabolomics of these children to further explore the metabolism of Chinese children with NAFLD.

The current study has some limitations in experimental design and data analysis. First, the number of children and adolescents studied was relatively small. It was difficult to restrict the same diet in all subjects, which may affect the composition and metabolism of gut microbiota. Second, we assessed gut microbiota via fecal samples instead of intestinal samples. It could not reflect all gut microbiota in the intestine exactly. However, lack of any non-invasive methods to directly obtain samples in the intestine could be the main cause. Therefore, we chose fecal samples to represent gut microbiota. Third, we did not evaluate serum metabolic markers as we described in a previous study (17). Further investigation is needed to assess the connection between gut microbiota and these serum factors.

## Conclusion

In conclusion, large variations of the composition and functional annotations of gut microbiota of NAFLD patients, obese, and healthy children exist in Chinese children and adolescents. The findings in our study and other studies are noteworthy in the understanding of gut microbiota in pediatric NAFLD. Further analysis is necessary to reveal the consistent relationship and molecular mechanism of gut microbiota in NAFLD pathogenesis.

## Data Availability Statement

The datasets generated for this study can be found in the Sequence Read Archive (https://www.ncbi.nlm.nih.gov/sra) under Bioproject PRJNA578215 and will be released upon the publication of this article.

## Ethics Statement

The studies involving human participants were reviewed and approved by Human Ethics Committee of Shenzhen Children's Hospital. Written informed consent to participate in this study was provided by the participants' legal guardian/next of kin.

## Author Contributions

SZ designed experiments. YZ, JZ, JL, MC, and SZ collected the data and performed the research. YZ, JZ, ZW, and SZ analyzed the data. YZ and MC wrote the manuscript. SZ critically reviewed and revised the manuscript for final submission.

### Conflict of Interest

The authors declare that the research was conducted in the absence of any commercial or financial relationships that could be construed as a potential conflict of interest.

## Abbreviations

BMI: body mass index
KEGG: kyoto encyclopedia of genes and genomes
KOs: KEGG orthologies
NAFLD: nonalcoholic fatty liver disease
NASH: nonalcoholic steatohepatitis
MRS: magnetic resonance spectroscopy
PDFF: proton density fat fraction.
