# Supplementary material for: Metagenome of Gut Microbiota of Children With Nonalcoholic Fatty Liver Disease
Source: Front Pediatr. 2019 Dec 20;7:518. doi: 10.3389/fped.2019.00518 (PMC6933441; doi:10.3389/fped.2019.00518)
Supplement: Supplementary file 1 [file Table_1.DOCX]

**Supplemental table 1. Abundant taxa of gut microbiota of all subjects**

| Phylum | Class | Order | Family | Genus | Species |
| --- | --- | --- | --- | --- | --- |
| Firmicutes | Negativicutes↑ | Selenomonadales↑ | Lactobacillaceae↓  Oscillospiraceae↓  Selenomonadaceae↑ | Faecalibacterium↓  Lactobacillus↓  Oscillibacter↓  Phascolarctobacterium↑  Ruminiclostridium↓ | Faecalibacterium prausnitzii  Phascolarctobacterium succinatutens↑  Ruminococcus callidus↓ |
| Bacteroidetes↓ | Bacteroidia↓ | Bacteroidales↓ | Odoribacteraceae↓  Rikenellaceae↓ | Alistipes↓  Odoribacter↓  Paraprevotella↓ | Bacteroides clarus↓  Odoribacter splanchnicus↓  Parabacteroides johnsonii↓ |
| Actinobacteria |  |  |  |  |  |
| Proteobacteria↑ | Gammaproteobacteria↑ | Enterobacterales↑ | Enterobacteriaceae↑  Helicobacteraceae | Helicobacter↓  Klebsiella↑  Kluyvera↑ | Helicobacter pylori↓  Klebsiella pneumoniae↑  Kluyvera ascorbata↑ |

↓ or ↑ representing abundance decrease or increase in obese children with and without NAFLD, compared to healthy controls
