# Supplementary material for: Metagenome of Gut Microbiota of Children With Nonalcoholic Fatty Liver Disease
Source: Front Pediatr. 2019 Dec 20;7:518. doi: 10.3389/fped.2019.00518 (PMC6933441; doi:10.3389/fped.2019.00518)
Supplement: Supplementary file 2 [file Data_Sheet_1.PDF]

伦理审查批件

批 件 号	深儿医伦审(科研)批件 2016020 号		
项目名称	儿童非酒精性脂肪肝病的肠道菌群基因分析		
项目来源	深圳市科技创新项目基础研究（自由探索）		
研究单位	深圳市儿童医院消化科		
主要研究者	周少明		
审查类别	初始审查	审查方式	快速审查
审查日期	2017.04.01	审查地点	新楼伦理办公室
审查委员	主审委员：邱宝明、胡雁		
批准文件	见附页		

审查意见：

根据卫生部《涉及人的生物医学研究伦理审查办法（试行（2007））》、SFDA《药物临床试验质量管理规范（2003）》、《医疗器械临床试验规定（2004）》、WMA《赫尔辛基宣言》和 CIOMS《人体生物医学研究国际道德指南》的伦理原则，经本伦理委员会审查，同意按所批准的临床研究方案、知情同意书、招募材料开展本项研究。

请遵循 GCP 原则、遵循伦理委员会批准的方案开展临床研究，保护受试者的健康与权利。

研究开始前，请申请人完成临床试验注册。

研究过程中，若变更主要研究者，对临床研究方案、知情同意书、招募材料等的任何修改，请申请人提交修正案审查申请。

发生严重不良事件时，请申请人及时提交严重不良事件报告。

请按照伦理委员会规定的年度和定期跟踪审查频率，申请人在截止日期前 1 个月提交研究进展报告；申办者应当向组长单位伦理委员会提交各中心研究进展的汇总报告；当出现任何可能显著影响试验进行或增加受试者危险的情况时，请申请人及时向伦理委员会提交书面报告。

研究纳入了不符合纳入标准或符合排除标准的受试者，符合中止试验规定，而未让受试者退出研究。给予错误治疗或计量，或给予方案禁止的合并用药等没有遵从方案开展研究的情况；或可能对受试者的权益和健康以及研究的科学性造成不良影响等违背 GCP 原则的情况，请申办者、监查员、研究者提交违背方案报告。

申请人暂停或提前终止临床研究，请及时提交暂停或终止研究报告。

完成临床研究，请申请人提交结题报告。

年度、定期跟踪审查频率	□3 个月 □6 个月 √1 年 □不适用
有效期	2017 年 04 月 01 日-2019 年 04 月 01 日
联系人与联系电话	联系人：祖莹，联系电话：0755-83008379
主任委员签字	[signature]
伦理委员会	深圳市儿童医院 伦理委员会（盖章）
日 期	2017 年 04 月 01 日

深圳市儿童医院医学伦理委员会制
